# Integrating Natural Language Processing and Interpretive Thematic Analyses to Gain Human-Centered Design Insights on HIV Mobile Health: Proof-of-Concept Analysis

**DOI:** 10.2196/37350

**Published:** 2022-07-21

**Authors:** Simone J Skeen, Stephen Scott Jones, Carolyn Marie Cruse, Keith J Horvath

**Affiliations:** 1 Department of Social, Behavioral, and Population Sciences School of Public Health and Tropical Medicine Tulane University New Orleans, LA United States; 2 Department of Psychology Hunter College City University of New York New York, NY United States; 3 Department of Psychology San Diego State University San Diego, CA United States

**Keywords:** mHealth, mobile health, HIV, natural language, thematic analysis, human-centered design, human-centered, user-centered, user-generated content, proof-of-concept, user feedback, web-based, web app, men's health, peer support, informal support, support group, digital health, eHealth, sentiment, design insight, user insight, Python, model, machine learning

## Abstract

**Background:**

HIV mobile health (mHealth) interventions often incorporate interactive peer-to-peer features. The user-generated content (UGC) created by these features can offer valuable design insights by revealing what topics and life events are most salient for participants, which can serve as targets for subsequent interventions. However, unstructured, textual UGC can be difficult to analyze. Interpretive thematic analyses can preserve rich narratives and latent themes but are labor-intensive and therefore scale poorly. Natural language processing (NLP) methods scale more readily but often produce only coarse descriptive results. Recent calls to advance the field have emphasized the untapped potential of combined NLP and qualitative analyses toward advancing user attunement in next-generation mHealth.

**Objective:**

In this proof-of-concept analysis, we gain human-centered design insights by applying hybrid consecutive NLP-qualitative methods to UGC from an HIV mHealth forum.

**Methods:**

UGC was extracted from Thrive With Me, a web app intervention for men living with HIV that includes an unstructured peer-to-peer support forum. In Python, topics were modeled by latent Dirichlet allocation. Rule-based sentiment analysis scored interactions by emotional valence. Using a novel ranking standard, the experientially richest and most emotionally polarized segments of UGC were condensed and then analyzed thematically in Dedoose. Design insights were then distilled from these themes.

**Results:**

The refined topic model detected K=3 topics: A: disease coping; B: social adversities; C: salutations and check-ins. Strong intratopic themes included HIV medication adherence, survivorship, and relationship challenges. Negative UGC often involved strong negative reactions to external media events. Positive UGC often focused on gratitude for survival, well-being, and fellow users’ support.

**Conclusions:**

With routinization, hybrid NLP-qualitative methods may be viable to rapidly characterize UGC in mHealth environments. Design principles point toward opportunities to align mHealth intervention features with the organically occurring uses captured in these analyses, for example, by foregrounding inspiring personal narratives and expressions of gratitude, or de-emphasizing anger-inducing media.

## Introduction

### Background

The advent of antiretroviral therapy (ART) marked an inflection point in the global AIDS epidemic, transforming HIV into a manageable chronic condition [1-3]. With people living with HIV who maintain undetectable viral loads incapable of passing the virus to their sexual partners, viral suppression by optimizing ART adherence is now a key tenet of population-level HIV-prevention planning [4,5]. However, ART adherence remains a challenge for many people living with HIV, endangering their health through viral rebound [6]. These challenges are attributable to a range of interlocking factors, many of them mirroring broader societal inequities in the United States: mistrust of medical providers, logistical and financial burdens of medical appointments, and stigma [7-10]. Unreliable transit, a lack of accessible brick-and-mortar services, and trauma can compound these challenges, particularly for many Black men who have sex with men (MSM) [11,12].

These persistent challenges suggest that traditional clinic-based treatment programs may be inadequate for fulfilling the needs of many MSM living with HIV. Mobile health (mHealth) interventions, which offer tools such as informational videos, hyperlocal service guides, and peer-support forums, have shown promise in this domain [13-17], including among MSM [15]. Many mHealth interventions include user-centered adaptations to bolster their appeal to user bases who inhabit intersecting identities (eg, Messages4Men for Black and Latino MSM [18]) or undertake specific risk behaviors (eg, APP+ for stimulant-using MSM [19]).

Traditional formative methods [20-23], often guided by the principles of user- and human-centered design (HCD [24-29]), aim to incorporate the insights of prospective mHealth user bases. Focus groups, user-experience interviews, and related in-person or virtual interactions are often undertaken to gain these insights. These methods can represent important contributions toward global health equity [30,31]. However, by relying on in-depth and often iterative interactions such as “think-aloud” usability tests [32], these methods can be burdensome to members of the communities they aim to empower, requiring time and logistical commitments akin to traditional study participation [33-35]. One alternative to these immersive approaches is mining user-generated content (UGC), comprising rich, unstructured, text-based data that end users themselves contribute to platforms, often in the form of social media posts or product reviews [36,37]. Across diverse sectors [37-39], UGC is increasingly recognized as an unmediated source of experiential data, through which consumers’, citizens’, and end users’ needs can be ascertained noninvasively at scale [40,41].

The scale of UGC data can introduce analytic challenges. The extraction of meaningful units of analysis among vast unstructured data is the foremost among those challenges [42]. Natural language processing (NLP) approaches, which rely on machine-readable elements such as keyword frequencies and probabilistic distributions of keyword clusters [43], are often employed for UGC analyses [44,45]. One common NLP technique is topic modeling (TM), in which the likelihood of contextually meaningful terms to co-occur in relative proximity to each other and thus signify a discrete topic within an unstructured text is computed [46]. For example, the relative proximity of the terms “epidemic,” “antiretroviral,” and “suppression” in the opening paragraphs of this introduction would be highly unlikely to occur by chance alone. Instead, their likelihood to co-occur in those passages can be interpreted as a meaningful signifier of the topic in those passages, namely HIV treatment. The topic model itself is composed of these co-occurring terms [43]. Another widely employed NLP technique, sometimes used in concert with TM [47], is sentiment analysis (SA). SA refers to a variety of tools that map individual keywords and other syntactic units to a prevalidated human-rated lexicon, computing a crude but summative account of the prevailing emotional tenor of a text [45,48].

NLP techniques are typically incapable of preserving narrative, subtext, and nuance [49,50]. Within digital health research, recent attempts to address these shortcomings have integrated NLP with traditional qualitative methods. These methods, although fruitful, remain exploratory, and are often resource-intensive, with little evident standardization in methods. In health sciences, combined NLP and qualitative approaches have been applied, preliminarily, toward cross-validation of each respective approach. For example, Leeson et al [51] have shown that conceptual overlaps among the findings of probabilistic TM using the Gensim toolkit in Python, the neural network application Word2Vec, and open qualitative coding are broad but not uniform [51], demonstrating the value of a “both-and” versus an “either-or” approach to machine- versus human-optimized analyses of UGC. The clearest strength of the “both-and” approach is its ability to analyze very large textual data sets, while preserving important nuance. To this end, Guetterman et al [52] combined qualitative coding and an NLP semantic-similarity clustering technique to classify open-ended text message responses to the MyVoice national youth poll. Through a modified 2-arm crossover experiment, NLP, qualitative, and sequential NLP-qualitative and qualitative-NLP variations were compared. Although the latter sequential approaches proved most time-consuming, they were able to check the validity of exploratory qualitative work or cultivate more nuanced interpretations of NLP-applied topics, respectively [51]. Jones et al [53] used a sequential qualitative-NLP approach to model topics across 4,901,516 posts contributed to 5 breast cancer forums scraped (with permission) from the open web. Timimi et al [54], examining UGC from the Inspire online support communities, used a nested NLP-qualitative approach to generate “entities” (a clustering technique) across more than 11 million unique posts. An inductive thematic coding analysis, applied to a subset of 246 posts, aided in developing a patient-centered lexicon to identify cognitive impairment side effects related to statin use.

Specifically, within mHealth, Petersen et al [55] integrated latent Dirichlet allocation (LDA) TM and SA with standard assessments of usability within a user-centered app design process. The sentiment of formative user interviews trended more positive as development progressed, which was reflected through improvements in the System Usability Scale (though not usefulness, satisfaction, and ease of use) scores. To our knowledge, no prior studies have applied a combined NLP-qualitative approach to textual UGC derived from an interactive mHealth environment. This is despite recent calls to bridge the respective strengths of data mining, at scale, with the richly realized insights provided by end-user narratives, to advance design practices in mHealth [56]. These detailed user-experience insights are necessary to advance mHealth design within the HCD paradigm [24,57,58]. If mHealth is to play a key role in the global HIV epidemic response, its persistent adoption will require deeply humanistic, yet scalable, strategies to guide user-centered adaptation. To this end, analyses of UGC in HIV mHealth must preserve the full range of human experiences and unique needs of multiply marginalized people living with HIV.

### Objectives

Recent findings point to the relative strengths of the sequential NLP-qualitative approach toward characterizing large-scale UGC, while preserving experiential nuance [51-55]. We applied a variation of this approach to UGC from the peer-support forum of Thrive With Me, a web app tailored for gay and bisexual MSM living with HIV [59,60]. Blending the strengths of machine-optimized techniques using NLP analyses with the strengths of traditional qualitative analyses, our findings were guided by the following aims:

Aim 1: To demonstrate the viability of a novel, sequential, NLP-qualitative approach toward characterizing UGC contributed by the end users of Thrive With Me

Aim 2: To examine the implications of the UGC-derived insights obtained in Aim 1 toward developing user-centered design adaptations for the next generation of HIV mHealth interventions

## Methods

### Study Intervention

**Figure 1:**
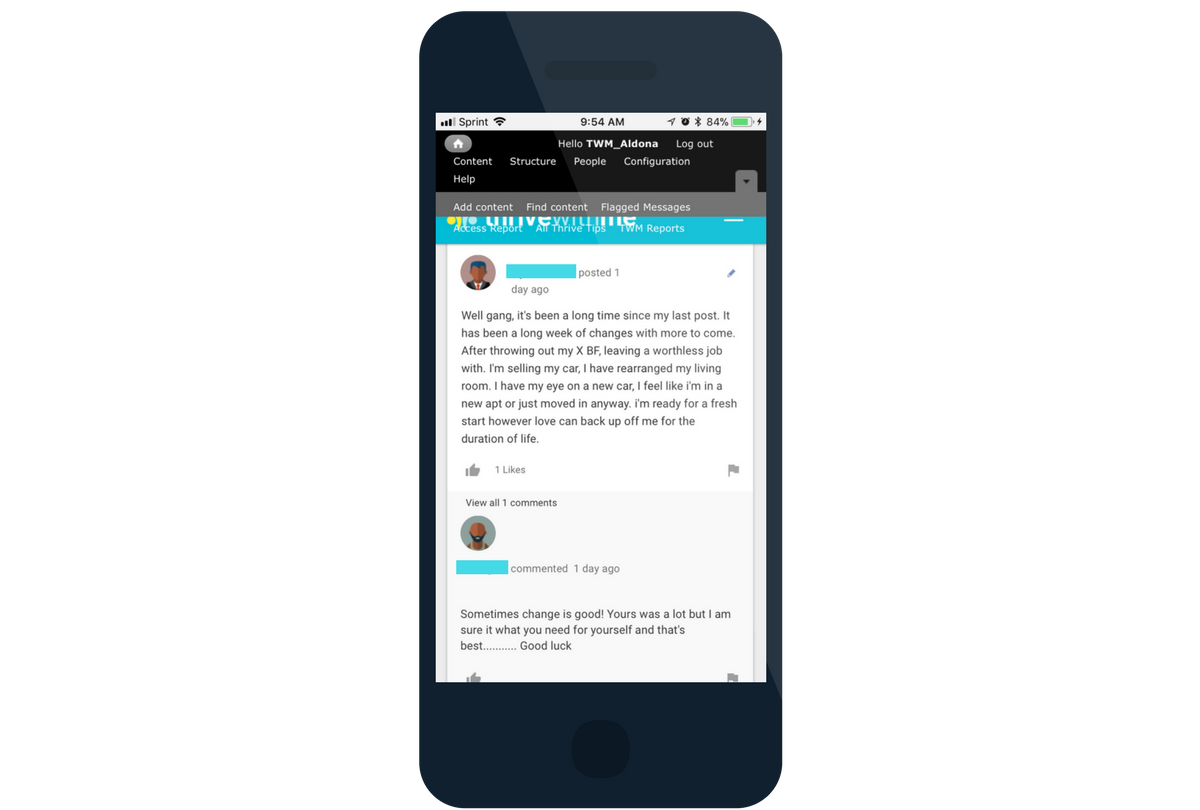
Illustrative screenshot of the Thrive With Me peer-support forum’s user interface. Posts and comments in the screenshot were mocked up by the study staff for demonstration purposes.

Thrive With Me is a web app–delivered intervention that combines self-monitoring tools for ART adherence, informative multimedia covering ART adherence, and asynchronous peer-to-peer support within a pseudonymous forum with the aim of improving treatment adherence among MSM living with HIV. Its components are grounded in the Information-Motivation-Behavioral skills (IMB) model of health behavior change [61]. An early iteration of Thrive With Me demonstrated preliminary efficacy versus treatment as usual in a pilot randomized controlled trial [59]. A prospective 2-arm randomized controlled trial testing a refined version of Thrive With Me versus an information-only control condition finished in 2019, with outcome analyses presently underway [60]. A screenshot of the user interface on which Thrive With Me users interacted is shown in Figure 1.

### Study Population

Participants were eligible if they (1) were HIV seropositive, (2) identified as males, (3) had a self-reported detectable viral load or suboptimal (<90%) ART adherence in the past 30 days, (4) reported sex with another man in the past 12 months, (5) could read and write English, (6) resided in the New York City area, and (7) had access to the internet and SMS text messaging for the duration of the study [60]. This study analyzed UGC contributed by participants randomized to the trial’s active intervention condition (N=202), who were given access to the Thrive With Me web app for a period of 5 months at baseline. (Throughout, we use “UGC” to refer to unstructured text exclusively, distinct from paradata or usage analytics.) The subsample’s sociodemographic attributes are shown in Table 1. Full details of the Thrive With Me parent trial are available elsewhere [60].

**Table 1:** Baseline characteristics of Thrive With Me study participants in the intervention arm.

Demographics				Thrive With Me intervention arm (N=202)
Age, mean (SD)				40.1 (10.8)
Male, n (%)				202 (100)
**Race, n (%)**				
	African American or Black			123 (61)
	American Indian/Alaskan Native			1 (0.5)
	Asian			1 (0.5)
	Native Hawaiian or Pacific Islander			2 (1.0)
	White			54 (27)
	More than one race			12 (5.9)
	Not reported			9 (4.5)
Hispanic**,** n (%)				62 (31)
**Education, n (%)**				
	High school or less			59 (29)
	Some college/associates/technical degree			90 (45)
	College/postgraduate/professional degree			52 (26)
	Not reported			1 (0.5)
**Employment status, n** **(%)**				
	Full-time			41 (20)
	Part-time			45 (22)
	Unemployed			77 (38)
	Disabled			35 (17)
	Retired			2 (1.0)
	Not reported			2 (1.0)
**Viral load (VL) measures**				
	**VL (biological) (<20), n (%)**			
		Detectable VL	74 (37)	
		Undetectable VL	127 (63)	
		Not reported	1 (0.5)	

### Ethics Approval

All study procedures and the use of associated data for secondary analyses were approved by the ethics review boards of the University of Minnesota (#1504S69721) and Hunter College of the City University of New York (#2015-0641).

### Procedures

**Figure 2:**
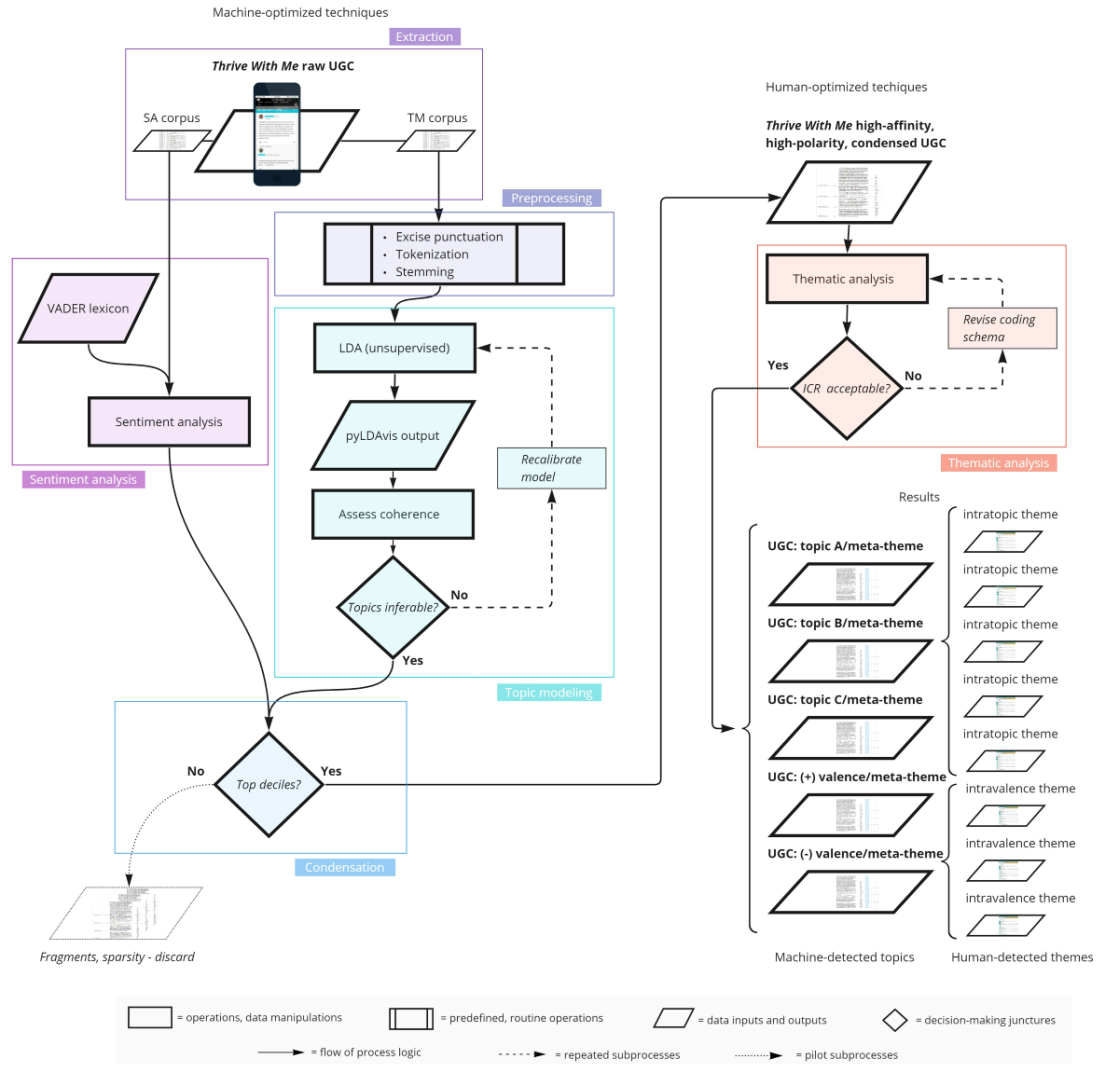
Flowchart of sequential machine- and human-optimized techniques. ICR: intercoder reliability; LDA: latent Dirichlet allocation; SA: sentiment analysis; TM: topic modeling; UGC: user-generated content; VADER: Valence Aware Dictionary for sEntiment Reasoner.

Initially, our procedures relied on the NLP techniques of unsupervised TM and rule-based SA to capture the semantic attributes of UGC drawn from Thrive With Me. We then employed a novel ranking technique to condense the richest and most emotionally polarized UGC. Finally, the detailed insights included in this condensed UGC were explored using the qualitative technique of interpretive thematic analysis. A flowchart of our complete procedure is shown in Figure 2.

#### Data Extraction

Textual UGC from Thrive With Me’s peer-support forums were extracted by the web app’s developer, Radiant, as a structured .csv file using the Drupal content management system’s Entity Export CSV function. Original posts and the comments they accrued were handled uniformly (referred to as posts throughout) for the sake of analysis. Content generated by study staff during prelaunch testing was removed manually before preprocessing. With test content excised, the raw UGC corpus contained 4912 posts and 147,649 total words. To accommodate necessary differentiation in the preprocessing steps, 2 UGC corpora were created: the SA corpus and TM corpus.

#### Data Preprocessing

All subsequent data preprocessing and NLP analyses were undertaken in Python (version 3.7.10, Python Software Foundation) on the Windows 10 (Microsoft Corporation) operating system.

In the TM corpus, first, unigram frequencies were calculated, and any unigrams occurring fewer than 3 times were discarded. The 571-term SMART (System for the Mechanical Analysis and Retrieval of Text) stop list was applied, excising all unigrams, such as “the” and “of,” terms whose co-occurrences are not typically indicative of the underlying topics from the raw TM corpus [62,63]. Capitalization and punctuation were removed throughout. All terms were converted to lowercase and then “split by whitespace” to ensure consonance in model inputs [43].

In the SA corpus, all semantic elements were preserved. In social media environments such as the Thrive With Me forum, peculiarities in syntax may amplify or even invert the intended sentiment of a text (eg, “so happy” versus “SOO happy!!! <3” versus “sooo happy. /s”) and thus represent important model inputs to retain [64].

#### TM Process

All steps in TM were applied to the TM corpus. We used the unsupervised LDA algorithm native to the scikit-learn (“sklearn”) Python library [65]. LDA is a generative probabilistic model that outputs a distribution of words (termed “tokens” [66]), which characterize the discrete topics within a text corpus [46]. K, the number of topics an LDA model will detect, is a model input determined based on prior familiarity with a corpus, relevant domain expertise, and the results of exploratory analyses [43]. Replication scripts for LDA TM are provided in Multimedia Appendix 1.

Each LDA model was evaluated for coherence by the first and second authors (SJS and SSJ) aided by the pyLDAvis tool. pyLDAvis plots modeled topics in 2 dimensions represented by circles, allowing for visual inspection of intertopic distances (how thematically distinct each topic is) and topic prevalence (how much content within a corpus each topic captures). A satisfactory K is characterized visually by circles with sufficiently large radii to capture a substantive share of a corpus and negligible overlap between circles, indicating discriminant interpretability across topics [67]. Detailed documentation on the use of pyLDAvis is available elsewhere [68]. We denote this preliminary LDA model as Model 1, which advanced to first-pass thematic analysis.

Finally, informed by Schofield and colleagues [62,63] and based on the coding schema developed inductively with Model 1, we removed high-frequency, non-topic–specific n-grams to generate a more intuitive set of tokens. This second pass was used to provide more self-evidently meaningful clusters of tokens for this proof-of-concept analysis. We denote this final model as Model 2.

Topic labels were developed based on domain knowledge, visual inspection of the top 30 per-topic tokens, and their particular distribution and contextual usage within the full series of posts assigned to each topic. Labels were finalized based on consensus between the first and second authors (SJS and SSJ).

#### SA Process

All steps in SA were applied to the SA corpus using the vaderSentiment library in Python [69]. We used the human-validated VADER (Valence Aware Dictionary for sEntiment Reasoner) sentiment lexicon, which scores the valence and intensity of individual terms and their related semantic elements, such as emoticons (“(: ”) and abbreviations common to social media and web-based forums (“lol” and “wtf”). VADER outputs polarity (positive-neutral-negative, on a scale of –1 to +1) scores for each input string [64]. For this analysis, we generated sentiment polarity and compound scores per unique post. As the richest instances of neutral-sentiment UGC were thematically redundant with the posts examined via LDA, we focused on emotionally polarized UGC captured by VADER’s positive and negative polarity scores. This focus on polarized UGC allowed us to explore sources and expressions of distress, while highlighting organically occurring positive interactions among Thrive With Me users.

Replication scripts for VADER SA are provided in Multimedia Appendix 1.

#### Condensation

Data condensation strengthens an analytic sample by honing it to its richest, most illustrative cases [70]. To condense the raw 4912-post UGC corpora, we used a novel percentile-ranking standard, loosely informed by (and considerably simplified from) the work of Nikolenko and colleagues [49] to advance the most meaningful data toward thematic analysis.

In the TM corpus, we calculated a simple affinity score for each post by summing the number of topic-specific tokens that appeared within that post. In this context, affinity refers to the degree to which each post is representative of the topic to which it has been assigned [49]. Using the =PERCENTILE() function in Excel (Microsoft Corporation) [71], we identified the 90th percentile affinity score for each topic, discarding posts that contained fewer topic-specific tokens than the 90th percentile thresholds.

In the SA corpus, we relied on VADER-generated polarity scores for percentile ranking. Posts that fell below the 90th percentile polarity score for positive and negative valences were discarded.

The SA and TM corpora were percentile-ranked independently. LDA modeling, which relies on co-occurrence of terms, favors verbose UGC, whereas VADER, reliant on purer expressions of sentiment, favors concision; hence, no UGC was duplicated in the condensed TM and condensed SA corpora. Specifically, richer and verbose UGC was emphasized in the condensed TM corpus, whereas emotive and concise UGC was emphasized in the condensed SA corpus.

A 90th percentile cutoff was chosen to condense a data set such that it became compact enough to be handled by 2 human coders (SJS and CMC) for the following inductive thematic analyses.

#### Interpretive Thematic Analyses

The condensed data set, comprising high-affinity and high-polarity UGC, was then subdivided into .csv files for thematic analysis by human coders. We used an inductive latent-level approach to examine the underlying concepts and discursive nuances intratopically [72]. Each stable topic and the strongest positively and negatively scored posts were thus handled as a meta-theme, each within a discrete .csv file. Human coders (SJS and CMC) undertook immersive close reads of these posts, identifying emergent intratopic themes and building pilot codes, first independently and then collaboratively, informed by the RADaR (rigorous and accelerated data reduction) technique in Excel [73]. Initially, we conducted open coding in Excel to leverage the accessibility of rapid matrix analysis techniques undertaken with nonspecialized software and to facilitate the necessary sorting and ranking of posts. Codes were applied iteratively and the overall coding schema was refined in conference, until unanimity in coding applications was obtained. Then, all data were migrated to Dedoose (SocioCultural Research Consultants) for final coding of the condensed data set that included LDA Model 1, where an overall pooled intercoder reliability of *κ*=0.78 was achieved [70,74]. Finally, after obtaining the acceptable intercoder reliability, the first author independently applied the coding schema to the condensed data set that included LDA Model 2 in Dedoose, producing the final coding applications reported here.

## Results

### TM Process

The LDA model rated for optimal coherence comprised K=3 topics, each composed of 30 co-occurring tokens. Topic A, disease coping [75], encompassed all posts in which the subject of living with HIV as a chronic condition predominated. Topic B, social adversities, covered those posts explaining the difficulties of navigating the interpersonal sphere as a person living with HIV. Topic C, salutations and check-ins, covered the broad array of brief greetings and personal updates routinely shared by users of the Thrive With Me forum. From the refined model, Model 2, our condensed data set included the 67 posts that contained more than 5 topic A–specific tokens (mean 7.31, SD 1.83), 118 posts containing more than 6 topic B–specific tokens (mean 9.43, SD 2.05), and 113 posts containing more than 4 topic C–specific tokens (mean 5.81, SD 1.14).

Altering the percentile split (whose primary rationale in this study was pragmatic) would have varied the size of the condensed UGC corpus considerably. In topic A, at the 75th percentile, at >3 tokens per post, 188 posts would be carried forward to thematic analysis; at the 95th percentile, or >6 tokens per post, 38 posts would be carried forward. In topic B, at the 75th percentile, at >4 tokens per post, 270 posts would be carried forward to thematic analysis; at the 95th percentile, or >8 tokens per post, 72 posts would be carried forward. Topic C, given the sparser nature of its UGC, was more dispersed. At the 75th percentile, at >2 tokens per post, 522 posts would be carried forward to thematic analysis; at the 99th percentile, or >6 tokens per post, only 20 posts would be carried forward.

The Model 2 tokens that characterize these topics, their labels and definitions, details of their condensation including the 90th percentile affinity score thresholds, and illustrative excerpts are shown in Table 2.

The number of posts and the number of tokens detected in LDA modeling by topic and by user are tabulated in Multimedia Appendix 2.

**Table 2:** Machine-detected topics, token n-grams, intratopic condensation, definitions, and illustrative examples.

Topic	Model 1 tokens	Model 2 tokens	Label	Definition		Model 2			
						Posts per topic, n (%) (N = 4912)	90th percentile threshold	High-affinity posts per topic, n (%) (N=1276)	Example high-affinity posts^a^
A	aids, care, com, doctor, don, effects, free, health, help, hiv, http, https, just, know, living, meds, need, new, people, positive, support, taking, thanks, time, took, treatment, undetectable, use, www, yes	aids, care, community, days, doctor, effects, feel, free, gay, health, hiv, living, know, meds, man, men, need, new, people, positive, really, sex, support, taking, think, time, took, treatment, undetectable, use	Disease coping	Portrayals of daily living with HIV, emphasizing serostatus awareness, ART^b^ regimens, and other sociomedical topics		1028 (20.92%)	>5 topic-specific tokens per post	67 (5.25%)	I don’t *think* disclosing an *HIV undetectable* viral load will persuade anyone who is *HIV* negative that we’re less likely to infect them. It can be *use*ful for potential partners that are also *HIV positive* beca*use* they are more likely to understand and accept that an *undetectable* viral load lowers the risk for re infection. Someone looking to avoid *HIV* or the risk of having *sex* with anyone *HIV* infected will likely not *care* or understand about *undetectable* viral loads.
B	blessed, cause, com, come, day, don, feel, gay, good, https, just, know, life, like, love, make, morning, men, real, really, people, person, sex, say, things, think, time, want, way, www	better, blessed, cause, come, day, feel, gay, good, hard, know, life, live, love, make, man, men, people, person, point, need, new, real, really, say, think, time, want, way, work, year	Social adversities		Portrayals of challenges and accomplishments in navigating sociality and sexuality as a sexual minority MSM living with HIV	1555 (31.65%)	>6 topic-specific tokens per post	118 (9.25%)	Truth b told…. what i am finding *hard* is to find guys that *want* more than a hook up....(they) al*way*s seem to *want* to sleep together FIRST (…) just sleeping with strangers right a*way* doesn’t turn me on like it used to...*make*s me *feel* kind of like a freak at *times*..... if all i *want*ed to do was ‘play’- I’d have NO problem finding guys to roll around with- even with my status- which i immediately and upfront disclose, both online and in *person*....... It’s finding guys who *want* conversation and dating and getting to *know* someone that has been *hard*est for me....
C	better, day, days, enjoy, feel, feeling, good, great, going, got, guys, happy, hey, hope, just, like, lol, man, morning, new, really, today, time, ve, welcome, year, years, week, weekend, work	best, better, day, doing, enjoy, feeling, going, good, got, great, guys, friday, happy, hello, Hey, hope, lol, luck, monday, morning, nice, really, sunday, thanks, time, today, week, weekend, welcome, wish	Salutations and check-ins	Greetings and brief personal updates		2329 (47.41%)	>4 topic-specific tokens per post	113 (8.86%)	*Morning* Thrivers! Can say much about my *weekend* cause I slept through it......... I just *wish* this holi*day* season would be over already so I can get back to some kind of normal being........ anyway I *wish* everyone a productive *week* and an *enjoy*able *thanks*giving..........

^a^The topic-specific tokens are italicized.

^b^ART: antiretroviral therapy.

^c^MSM: men who have sex with men.

### SA Process

For the positively valenced ([+]Pos) posts, our condensed data set included the 488 posts assigned a polarity score >0.659 by the VADER lexicon ([+]Pos intravalence mean 0.81, SD 0.12). For the negatively valenced ([–]Neg) posts, our condensed sample included the 490 posts that were assigned a polarity score >0.196 ([–]Neg intravalence mean 0.34, SD 0.16).

Details of the intravalence condensation of the strongly positive and negative posts, with illustrative examples, are shown in Table 3.

**Table 3:** VADER (Valence Aware Dictionary for sEntiment Reasoner)-assigned sentiment polarity, intravalence condensation, and illustrative examples.

Sentiment polarity	90th percentile threshold	High-affinity posts per valence, n (%) (N=1276)	Example high-affinity posts (including polarity scores)
(+)Pos^a^	>0.659 (+) score	488 (38.24%)	“Beautiful story, thanks for sharing” (0.828 Pos, 0.172 Neg)
			“I love you positiveness.............” (0.789 Pos, 0.000 Neg)
(–)Neg^b^	>0.196 (–) score	490 (38.4%)	“I hate trump (lower case)!!!” (0.000 Pos, 0.604 Neg)
			“Bad anxiety today. Even my blood pressure was high.” (0.000 Pos, 0.552 Neg)

^a^Positively valenced.

^b^Negatively valenced.

### Thematic Analyses

The condensed data set contained 1276 posts: 298 associated with the 90th percentile of the affinity of topics A, B, and C from LDA Model 2, and 978 associated with the 90th percentile of positive and negative polarity. This data set was advanced to thematic analysis. The detected intratopic and intravalence themes, their operational definitions, code co-occurrences, and illustrative excerpts are displayed in a meta-matrix in Multimedia Appendix 3.

Within topic A, most themes articulated the distinct, day-to-day obligations of living with HIV. The most frequently detected themes, which reflected informative prompts provided by the Thrive With Me web app, covered ART medications. These instances were rich enough to warrant the coding of dedicated subthemes capturing detailed adherence tips, personal antiretroviral regimens, and adverse effects. Issues of long-term survival were raised, as were various personal narratives and peer-to-peer recommendations for disclosing one’s HIV serostatus to potential sexual partners. Further, a code (“Raising Awareness”) captured the many instances in which users shared details of activist events, local resources, and HIV-tailored public health messaging.

Within topic B, diverse personal narratives were shared, including all articulations of the specific challenges that sexual minority MSM living with HIV may encounter as they seek social and sexual bonds with other men. These included mismatched expectations around relationship longevity and extradyadic pairings, life chaos attributed to partners’ alcohol and crystal meth use, and the roles of ex-partners. Trust, broken trust, discussions of self-confidence, and expressions of loneliness and isolation were emergent intratopic themes. The roles of support networks, including direct appeals to and provision of peer-to-peer social support among Thrive With Me users also emerged within this topic.

Within topic C, the overwhelming proportion of UGC was made of brief greetings. In posts where these greetings were expanded to include personal updates and peer check-ins, 2 intratopic themes predominated; the first comprised substance use, misuse, and recovery, which included disclosures of relapse among Thrive With Me users; second, an emergent theme of personal triumph was also evident, which covered accomplishments such as new physical fitness regimens, career successes, and the attainment of treatment goals such as stable CD4 counts.

Strongly positive posts were characterized by gratitude, typically in response to peer-to-peer encouragements and affirmations occurring on the forum. Strongly negative posts were richer and more thematically heterogenous. Many of these posts were reactions to linked external news media, which overwhelmingly provoked anger. These media often covered acts of homonegativity and racism. Another (–)Neg theme encompassed the political climate in the United States during the period of the Thrive With Me trial, when the 2016 presidential election was decided. The final intravalence theme concerned mental health, typically through expressions of acute or ongoing struggles with depression, stress, and insomnia.

## Discussion

### Principal Findings

We combined common NLP techniques with traditional latent thematic analysis to classify UGC drawn from an interactive HIV mHealth environment. Through multiple iterations of LDA modeling, stable topics emerged: the day-to-day concerns of living with HIV; the social, romantic, and sexual tolls of aging with HIV as a sexual minority MSM; and routine greetings and daily affirmations. Using a 90th percentile cutoff, we condensed the UGC of which these topics were composed from a total of 4912 posts to a rich, illustrative subset of 1276 posts. By further analyzing this condensed UGC as a set of meta-themes, we identified latent discourses within them, through which experiential design insights could be mined.

Our work contributes to the diverse, cross-disciplinary literature exploring sequential NLP-qualitative methods [49,51-55,76], while responding to the call of Britt and colleagues [56] to explore the possibilities of integrated data mining and narrative analyses in mHealth. By sequentially combining NLP and qualitative techniques, our work resembles recent analyses that demonstrated the ability of consecutive NLP-qualitative methods to create machine-generated meta-themes from web-based forum and text message data and, in turn, preserve narrative and context through qualitative coding [51-55]. In contrast to these analyses, we used UGC derived from an interactive mHealth environment, focusing on user-centered product adaptation as a potential application. In emphasizing design applications, our work resembles that of Petersen et al [55], who applied similar NLP techniques to interviews of prospective users of an exercise-promoting wearable technology, capturing improvements in sentiment and usability at 0-, 5-, and 10-week intervals. Unlike our own, this analysis [55] fulfilled the iterative criteria of a user-centered design cycle [22,26,27], forgoing the more labor-intensive aspects of qualitative analysis [70], while demonstrating its NLP-aided, user-centered approach.

To that end, the results reported here offer partial fulfillment of Aim 1. Although the sequential methods we demonstrated did successfully characterize the prevailing themes of the peer forum, the future viability of these methods will depend on their routinization. Our procedures included a number of transformations and cross-platform migrations, each of which introduces friction, which in turn disincentivizes adoption [77]. Routine NLP-enabled mHealth monitoring would, instead, require integrated text analytics [78,79] and graphical user interfaces to ensure accessibility for investigators without coding expertise [56]. Such “no-code” (a common industry term) solutions could aid in bridging the knowledge-translation gap through evidence synthesis and translation, a lasting challenge in implementation science [80], as well as in clinically integrating mHealth interventions [81]. Alternately, although our method demonstrates the value of maintaining human interpretability of NLP outputs, the very thematic codes we developed inductively might lend themselves, in future, to repurposing as target labels in training HIV-domain data sets for supervised deep-learning applications [82]. The inherent potential of such “both-and” approaches remains to be explored.

As for Aim 2, a range of actionable design insights surfaced from these findings to guide future iterations of Thrive With Me specifically and HIV mHealth generally. An HCD approach typically reframes these insights as “how might we” (HMW) prompts, a reframing we embrace here [24,26]. First, the seropositive MSM end users of Thrive With Me who engaged in the peer-support forum typically did so transparently and intimately, tapping their peers for encouragement, collaboratively navigating difficult subjects. These instances are most evident throughout topics A and B, specifically within the ART-related, “survivorship,” and “partnering challenges” themes and in the peer-to-peer affirmations surfaced via the (+)Pos UGC. Nevertheless, the forum was also, more problematically, a platform to express outrage at external news media. These media often recounted instances of homonegative violence and discrimination. These issues were, of course, clearly relevant to Thrive With Me users, as “reacting to media” codes, emergent within the (–)Neg condensed UGC (exclusively), occurred at twice the frequency of any other, with the exception of “partnering challenges” within topic B. However, their intrusive nature and negativity may have dampened the overall emotional tenor of the forum. These appeals to outrage may have discouraged newly enrolled or “lurking” users from interacting with the forum or disproportionately consumed their attention. In either scenario, the intended benefits of the social support provided by the forum may have been undermined. As such, HMW 1 is “How might future iterations of Thrive With Me acknowledge the anger evoked by an oppressive society without compromising the supportive aims of the peer forum?” Active content moderation, dedicated channels for current events, or even an embargo on outbound links might accomplish such an aim; however, these solutions would require prototyping and prospective end-user feedback in an HCD cycle [24].

Another topic, with several related intratopic themes, concerned relationship difficulties. In addition to the abovementioned “partnering challenges” theme emergent within topic B, unmet relationship needs were evident throughout the “trust and betrayal” and social isolation–focused “voids in my life” themes. Thus, HMW 2 is “How might we support the interpersonal needs of seropositive MSM without imposing model drift into an ART adherence intervention?” The latent need is evident, and the deliberations of end users often touched on cross-cutting topic A and (–)Neg themes; the richest instances of intertopic cross-codings are shown among the “disclosing serostatus” (topic A), “partnering challenges” (topic B), and “substance use and misuse” (topic C) themes, illustrating the entanglement of these issues in Thrive With Me users’ lives. Dedicated informational modules might address these needs more directly, tying decision-making within this domain to specific triggers for illegal drug use or missed ART doses in a manner consistent with the IMB model in which Thrive With Me is grounded [60,61].

Finally, a desire to narrativize the personal triumphs of HIV survivorship is often evident across topics A and C, particularly within the “survivorship” and (in vivo) “other days I move mountains” codes. These narratives, which cover grief, coming out, and the lessons imparted by long-term survival, surface as an organically occurring form of UGC, pointing out their importance to Thrive With Me users, perhaps as validations of their personal resilience. Such strength-based, person-centered affirmations may hold the potential to constructively reauthor Thrive With Me users’ experiences of societal oppressions, while finding resonances within each other’s stories [83,84]. If implemented carefully, such reframing may redirect the negativity discussed in HMW 1 without invalidating the stressors that drive it, while simultaneously encouraging engagement with the peer forum. An appropriate HMW 3 is “How might we activate the potential of personal narratives toward the well-being of MSM living with HIV?” Asynchronous health recovery narratives, even those scraped from UGC on the open web, can enhance behavior-change self-efficacy and the likelihood of cancer screening [85,86]. The curation of these narratives in a dedicated portal, akin to innovations in digital psychiatry such as the NEON (Narrative Experiences Online) intervention [87], might represent an adaptable periphery of next-generation HIV mHealth [88].

### Limitations

These findings are subject to a range of limitations. As a proof-of-concept analysis, our methods are exploratory. Nevertheless, the abovementioned migrations and transformations built into our methods allow for the imposition of human error, while rate-limiting the rapidity with which results can be generated. In contrast, the adoption of a single alternative developer environment such as R (R Foundation for Statistical Computing), which permits qualitative analyses via the R qualitative data analysis package [89] would enhance efficiency considerably. We were also limited by our inability to member-check our LDA modeling and thematic coding schema with Thrive With Me users themselves, which would have bolstered transactional validity and laid the groundwork for a true HCD process, incorporating iterative prototyping, design sprints, and feedback elicited from the user base whose needs we attempt to fulfill. The use of domain-expert raters employed by Nikolenko and colleagues [49] to assure the coherence and human interpretability of LDA outputs offers a template for such a member-checking approach. A de facto tension exists between HCD, which is nimble, creativity-driven, and interactive, and UGC analysis, which is static and typically archival. Innovative solutions, such as real-time syndromic surveillance on social media [90,91], point toward the possibilities of resolving this tension and toward potential innovations in interactive mHealth. Finally, through a design justice lens [31], we recognize that the approach we describe leverages analytic advancements undertaken in English, using English-language corpora, within an intervention context that requires users to receive information and interact in English [60]. Although the need for multilingual NLP is recognized within the field, progress remains limited [92]. Monolingual approaches toward capturing user-experience insights will, of course, remain narrow in scope amid the vast diversity of human speech.

### Conclusions

mHealth interventions that fulfill the needs of multiply marginalized MSM living with HIV must accommodate a diverse array of needs and experiences. The findings of this proof-of-concept analysis suggest that combined machine- and human-optimized techniques can capture actionable insights on these needs and experiences without adding to the burdens of prospective end users. By maintaining an empathic lens and focusing on refinements in method, techniques such as those demonstrated here can contribute to future innovations in HIV mHealth.

## Appendix

Multimedia Appendix 1 Text preprocessing, latent Dirichlet allocation topic modeling, and VADER (Valence Aware Dictionary for sEntiment Reasoner) sentiment analysis replication scripts in Python.

Multimedia Appendix 2 Tokens detected per user, per topic (Model 2).

Multimedia Appendix 3 Human-detected intratopic (Model 2) and intravalence themes with definitions and illustrative examples.

## Abbreviations

ART: antiretroviral therapy
HCD: human-centered design
HMW: how might we
IMB: Information-Motivation-Behavioral skills
LDA: latent Dirichlet allocation
mHealth: mobile health
MSM: men who have sex with men
(–)Neg: negatively valenced
NLP: natural language processing
(+)Pos: positively valenced
SA: sentiment analysis
TM: topic modeling
UGC: user-generated content
VADER: Valence Aware Dictionary for sEntiment Reasoner
