# Supplementary material for: Integrating Natural Language Processing and Interpretive Thematic Analyses to Gain Human-Centered Design Insights on HIV Mobile Health: Proof-of-Concept Analysis
Source: JMIR Hum Factors. 2022 Jul 21;9(3):e37350. doi: 10.2196/37350 (PMC9353680; doi:10.2196/37350)
Supplement: Multimedia Appendix 2 [file humanfactors_v9i3e37350_app2.docx]

**Multimedia Appendix 2.** Tokens detected per user, per topic (Model 2).

| PID | **Topic A** | | | **Topic B** | | | **Topic C** | | |
| --- | --- | --- | --- | --- | --- | --- | --- | --- | --- |
|  | *n* posts | Tokens detected | | *n* posts | Tokens detected | | *n* posts | Tokens detected | |
|  |  | *M (SD)** | *Mdn (IQR)* |  | *M (SD)* | *Mdn (IQR)* |  | *M (SD)* | *Mdn (IQR)* |
| 50032 | 11 | 1.55 (1.37) | 2.00 (0.00-3.00) | 14 | 1.79 (1.19) | 2.00 (1.00-3.00) | 17 | 1.35 (1.17) | 1.00 (0.00-2.00) |
| 50039 | 0 | — | — | 1 | 1.00 (0.00) | 1.00 (1.00-1.00) | 4 | 1.75 (1.26) | 2.00 (1.00-2.50) |
| 50041 | 0 | — | — | 0 | — | — | 0 | — | — |
| 50043 | 59 | 1.39 (1.56) | 1.00 (0.00-2.00) | 71 | 2.00 (2.22) | 1.00 (0.00-3.00) | 201 | 1.41 (1.19) | 1.00 (1.00-2.00) |
| 50046 | 0 | — | — | 0 | — | — | 2 | 2.00 (0.00) | 2.00 (2.00-2.00) |
| 50053 | 27 | 1.85 (1.76) | 1.00 (0.00-3.00) | 39 | 2.13 (1.87) | 2.00 (1.00-3.00) | 38 | 1.05 (1.06) | 1.00 (0.00-2.00) |
| 50055 | 20 | 2.65 (1.63) | 3.00 (1.00-4.00) | 26 | 2.54 (2.44) | 2.00 (1.00-4.00) | 10 | 1.50 (1.27) | 1.00 (1.00-2.00) |
| 50058 | 0 | — | — | 0 | — | — | 1 | 2.00 (0.00) | 2.00 (2.00-2.00) |
| 50059 | 19 | 0.84 (0.96) | 1.00 (0.00-2.00) | 14 | 2.57 (1.65) | 2.00 (1.00-4.00) | 112 | 3.12 (2.12) | 3.00 (1.00-5.00) |
| 50060 | 0 | — | — | 0 | — | — | 1 | 0.00 (0.00) | 0.00 (0.00-0.00) |
| 50071 | 16 | 2.88 (2.25) | 2.00 (1.00-4.00) | 23 | 2.87 (3.22) | 2.00 (0.00-4.00) | 36 | 1.67 (1.41) | 1.5 (0.00-3.00) |
| 50073 | 1 | 1.00 (0.00) | 1.00 (1.00-1.00) | 1 | 11.00 (0.00) | 11.00 (11.00-11.00) | 4 | 0.75 (1.50) | 0.00 (1.50-3.00) |
| 50080 | 2 | 1.00 (1.41) | 1.00 (0.00-2.00) | 1 | 0.00 (0.00) | 0.00 (0.00-0.00) | 1 | 2.00 (0.00) | 2.00 (2.00-2.00) |
| 50093 | 5 | 1.40 (1.52) | 1.00 (1.00-1.00) | 7 | 0.57 (0.79) | 0.00 (0.00-1.00) | 8 | 1.00 (0.93) | 1.00 (0.00-2.00) |
| 50095 | 2 | 5.00 (1.41) | 5.00 (4.00-6.00) | 1 | 5.00 (0.00) | 5.00 (5.00-5.00) | 6 | 2.17 (1.17) | 2.00 (1.00-3.00) |
| 50102 | 0 | — | — | 3 | 2.67 (3.06) | 2.00 (0.00-6.00) | 4 | 0.50 (0.58) | 0.50 (0.00-1.00) |
| 50113 | 0 | — | — | 0 | — | — | 4 | 1.75 (1.26) | 2.00 (1.00-2.50) |
| 50116 | 3 | 0.67 (1.15) | 0.00 (0.00-2.00) | 3 | 0.67 (0.58) | 1.00 (0.00-1.00) | 1 | 3.00 (0.00) | 3.00 (3.00-3.00) |
| 50128 | 2 | 2.00 (1.41) | 2.00 (1.00-3.00) | 1 | 2.00 (0.00) | 2.00 (2.00-2.00) | 1 | 1.00 (0.00) | 0.00 (0.00-0.00) |
| 50138 | 0 | — | — | 0 | — | — | 3 | 3.00 (1.00) | 3.00 (2.00-4.00) |
| 50145 | 1 | 0.00 (0.00) | 0.00 (0.00-0.00) | 0 | — | — | 7 | 2.00 (0.58) | 2.00 (2.00-2.00) |
| 50146 | 2 | 3.00 (0.00) | 3.00 (3.00-3.00) | 2 | 2.00 (1.41) | 2.00 (1.00-3.00) | 6 | 3.83 (2.40) | 4.00 (2.00-6.00) |
| 50147 | 0 | — | — | 1 | 5.00 (0.00) | 5.00 (5.00-5.00) | 1 | 4.00 (0.00) | 4.00 (4.00-4.00) |
| 50154 | 0 | — | — | 0 | — | — | 1 | 0.00 (0.00) | 0.00 (0.00-0.00) |
| 50182 | 0 | — | — | 0 | — | — | 3 | 0.33 (0.58) | 0.00 (0.00-1.00) |
| 50192 | 0 | — | — | 0 | — | — | 2 | 0.50 (0.71) | 0.50 (0.00-1.00) |
| 50193 | 76 | 1.18 (1.39) | 1.00 (0.00-2.00) | 105 | 1.28 (1.58) | 1.00 (0.00-2.00) | 122 | 1.05 (1.10) | 1.00 (0.00-2.00) |
| 50195 | 26 | 2.00 (2.19) | 1.00 (0.00-4.00) | 46 | 2.22 (2.93) | 1.00 (0.00-3.00) | 56 | 1.54 (1.54) | 1.00 (0.00-2.00) |
| 50209 | 5 | 2.60 (2.51) | 3.00 (0.00-5.00) | 2 | 0.50 (0.71) | 0.50 (0.00-1.00) | 4 | 2.25 (0.50) | 2.00 (2.00-2.50) |
| 50226 | 1 | 0.00 (0.00) | 0.00 (0.00-0.00) | 0 | — | — | 0 | — | — |
| 50234 | 4 | 2.00 (1.15) | 2.00 (1.00-3.00) | 0 | — | — | 4 | 1.25 (0.96) | 1.50 (0.50-2.00) |
| 50245 | 0 | — | — | 0 | — | — | 2 | 0.00 (0.00) | 0.00 (0.00-0.00) |
| 50256 | 0 | — | — | 0 | — | — | 6 | 1.16 (0.41) | 1.00 (1.00-1.00) |
| 50268 | 30 | 1.00 (1.58) | 1.00 (0.00-2.00) | 30 | 1.57 (1.81) | 1.00 (0.00-3.00) | 103 | 1.20 (1.24) | 1.00 (0.00-2.00) |
| 50278 | 0 | — | — | 7 | 3.14 (2.61) | 4.00 (0.00-5.00) | 6 | 3.50 (2.59) | 3.00 (2.00-6.00) |
| 50287 | 0 | — | — | 0 | — | — | 1 | 0.00 (0.00) | 0.00 (0.00-0.00) |
| 50288 | 0 | — | — | 0 | — | — | 1 | 2.00 (0.00) | 2.00 (2.00-2.00) |
| 50297 | 48 | 1.85 (2.39) | 1.00 (0.00-3.00) | 57 | 1.42 (1.76) | 1.00 (0.00-2.00) | 196 | 1.65 (1.32) | 1.00 (1.00-2.50) |
| 50367 | 0 | — | — | 0 | — | — | 2 | 2.00 (0.00) | 2.00 (2.00-2.00) |
| 50369 | 1 | 0.00 (0.00) | 0.00 (0.00-0.00) | 0 | — | — | 3 | 2.33 (0.58) | 2.00 (2.00-3.00) |
| 50371 | 3 | 4.00 (3.46) | 2.00 (2.00-8.00) | 7 | 3.86 (3.98) | 4.00 (0.00-6.00) | 3 | 1.67 (1.15) | 1.00 (1.00-3.00) |
| 50377 | 12 | 2.50 (1.57) | 2.00 (1.50-3.50) | 7 | 2.29 (1.80) | 1.00 (0.00-4.00) | 16 | 3.25 (2.18) | 3.00 (2.00-4.50) |
| 50413 | 0 | — | — | 1 | 1.00 (0.00) | 1.00 (1.00-1.00) | 1 | 2.00 (0.00) | 2.00 (2.00-2.00) |
| 50417 | 16 | 0.50 (1.10) | 0.00 (0.00-0.50) | 48 | 0.79 (1.18) | 0.00 (0.00-1.50) | 72 | 0.67 (0.99) | 0.00 (0.00-1.00) |
| 50442 | 0 | — | — | 1 | 1.00 (0.00) | 1.00 (1.00-1.00) | 0 | — | — |
| 50443 | 7 | 1.43 (2.23) | 0.00 (0.00-2.00) | 5 | 0.00 (0.00) | 0.00 (0.00-0.00) | 19 | 0.89 (1.20) | 1.00 (0.00-1.00) |
| 50445 | 2 | 0.00 (0.00) | 0.00 (0.00-0.00) | 1 | 0.00 (0.00) | 0.00 (0.00-0.00) | 3 | 2.00 (2.00) | 2.00 (0.00-4.00) |
| 50446 | 0 | — | — | 1 | 4.00 (0.00) | 4.00 (4.00-4.00) | 0 | — | — |
| 50454 | 0 | — | — | 0 | — | — | 1 | 2.00 (0.00) | 2.00 (2.00-2.00) |
| 50458 | 8 | 2.00 (1.77) | 2.00 (0.50-3.00) | 8 | 3.86 (3.23) | 3.5 (1.00-5.50) | 10 | 1.10 (0.99) | 1.00 (0.00-2.00) |
| 50459 | 1 | 2.00 (0.00) | 2.00 (2.00-2.00) | 0 | — | — | 1 | 5.00 (0.00) | 5.00 (5.00-5.00) |
| 50474 | 0 | — | — | 0 | — | — | 2 | 1.00 (0.00) | 1.00 (1.00-1.00) |
| 50481 | 53 | 1.58 (1.23) | 1.00 (0.00-2.00) | 84 | 2.60 (1.98) | 2.50 (1.00-3.50) | 62 | 1.63 (1.24) | 1.00 (1.00-2.00) |
| 50493 | 12 | 1.75 (1.91) | 1.00 (1.00-2.5) | 16 | 2.44 (1.55) | 2.50 (1.00-4.00) | 25 | 1.76 (1.27) | 2.00 (1.00-2.00) |
| 50498 | 14 | 1.71 (1.82) | 1.00 (1.00-2.00) | 29 | 2.45 (2.51) | 2.00 (2.00-4.00) | 113 | 1.77 (1.33) | 2.00 (1.00-2.00) |
| 50501 | 0 | — | — | 0 | — | — | 2 | 1.00 (1.41) | 2.00 (0.00-2.00) |
| 50520 | 30 | 1.47 (1.61) | 1.00 (0.00-2.00) | 41 | 2.32 (2.31) | 1.00 (1.00-3.00) | 86 | 1.34 (1.07) | 1.00 (1.00-2.00) |
| 50563 | 0 | — | — | 0 | — | — | 13 | 4.00 (1.00) | 4.00 (4.00-4.00) |
| 50591 | 1 | 0.00 (0.00) | 0.00 (0.00-0.00) | 9 | 2.22 (1.99) | 2.00 (1.00-3.00) | 4 | 1.00 (0.00) | 1.00 (1.00-1.00) |
| 50628 | 26 | 2.92 (2.17) | 2.00 (1.00-4.00) | 77 | 2.83 (2.65) | 2.00 (0.00-4.00) | 3 | 2.33 (1.53) | 2.00 (1.00-4.00) |
| 50637 | 52 | 2.41 (2.35) | 2.00 (1.00-4.00) | 0 | — | — | 100 | 2.05 (1.79) | 2.00 (1.00-3.00) |
| 50658 | 0 | — | — | 0 | — | — | 1 | 4.00 (0.00) | 4.00 (4.00-4.00) |
| 50660 | 0 | — | — | 4 | 3.00 (1.41) | 2.50 (2.00-4.00) | 3 | 4.00 (0.00) | 4.00 (4.00-4.00) |
| 50674 | 0 | — | — | 0 | — | — | 2 | 2.00 (0.00) | 2.00 (2.00-2.00) |
| 50690 | 2 | 1.00 (0.00) | 1.00 (1.00-1.00) | 2 | 0.50 (0.71) | 0.50 (0.00-1.00) | 4 | 1.50 (1.00) | 1.00 (1.00-2.00) |
| 50724 | 1 | 1.00 (0.00) | 1.00 (1.00-1.00) | 4 | 1.50 (1.73) | 1.00 (0.50-2.50) | 18 | 0.94 (0.54) | 1.00 (1.00-1.00) |
| 50726 | 0 | — | — | 1 | 0.00 (0.00) | 0.00 (0.00-0.00) | 0 | — | — |
| 50738 | 0 | — | — | 0 | — | — | 1 | 1.00 (0.00) | 1.00 (1.00-1.00) |
| 50773 | 9 | 1.11 (1.45) | 0.00 (0.00-3.00) | 5 | 1.60 (1.82) | 1.00 (0.00-3.00) | 18 | 1.28 (1.27) | 1.00 (0.00-2.00) |
| 50798 | 2 | 0.50 (0.71) | 0.50 (0.00-1.00) | 6 | 5.17 (2.56) | 4.50 (3.00-7.00) | 13 | 2.15 (1.28) | 2.00 (1.00-3.00) |
| 50810 | 0 | — | — | 0 | — | — | 6 | 1.00 (1.26) | 0.50 (0.00-2.00) |
| 50821 | 5 | 2.40 (2.30) | 3.00 (0.00-4.00) | 7 | 1.43 (1.27) | 1.00 (0.00-3.00) | 7 | 1.57 (1.27) | 2.00 (0.00-2.00) |
| 50848 | 32 | 0.63 (0.83) | 0.00 (0.00-1.00) | 44 | 0.91 (1.27) | 0.00 (0.00-1.00) | 74 | 1.22 (1.15) | 1.00 (0.00-2.00) |
| 50867 | 1 | 2.00 (0.00) | 2.00 (2.00-2.00) | 0 | — | — | 0 | — | — |
| 50869 | 9 | 0.44 (0.73) | 0.00 (0.00-1.00) | 12 | 0.83 (1.11) | 0.00 (0.00-2.00) | 67 | 1.75 (1.43) | 1.00 (1.00-2.00) |
| 50882 | 54 | 1.61 (1.58) | 1.00 (0.00-2.00) | 117 | 2.52 (2.19) | 2.00 (1.00-4.00) | 184 | 1.85 (1.21) | 2.00 (1.00-3.00) |
| 50884 | 5 | 1.20 (1.30) | 1.00 (0.00-2.00) | 8 | 3.25 (2.12) | 3.5 (1.50-5.00) | 24 | 1.79 (1.56) | 2.00 (0.00-3.00) |
| 50922 | 2 | 4.00 (2.83) | 4.00 (2.00-6.00) | 3 | 4.00 (1.00) | 4.00 (3.00-5.00) | 1 | 0.00 (0.00) | 0.00 (0.00-0.00) |
| 50927 | 1 | 0.00 (0.00) | 0.00 (0.00-0.00) | 4 | 1.00 (0.82) | 1.00 (0.50-1.50) | 0 | — | — |
| 50936 | 3 | 0.00 (0.00) | 0.00 (0.00-0.00) | 1 | 1.00 (0.00) | 1.00 (1.00-1.00) | 2 | 1.00 (1.41) | 1.00 (0.00-2.00) |
| 50954 | 5 | 5.40 (4.64) | 2.00 (0.00-11.00) | 4 | 8.50 (2.38) | 8.5 (5.50-10.50) | 3 | 3.33 (2.89) | 5.00 (0.00-5.00) |
| 50974 | 2 | 0.50 (0.71) | 0.50 (0.00-1.00) | 5 | 3.20 (3.22) | 3.00 (1.00-4.00) | 2 | 3.50 (2.12) | 3.5 (2.00-5.00) |
| 50986 | 7 | 3.14 (2.73) | 3.00 (0.00-6.00) | 2 | 0.50 (0.71) | 0.50 (0.00-1.00) | 1 | 2.00 (0.00) | 2.00 (2.00-2.00) |
| 50997 | 2 | 0.50 (0.71) | 0.50 (0.00-1.00) | 2 | 0.00 (0.00) | 0.00 (0.00-0.00) | 38 | 2.34 (1.12) | 2.00 (2.00-3.00) |
| 51022 | 0 | — | — | 1 | 1.00 (0.00) | 1.00 (1.00-1.00) | 1 | 2.00 (0.00) | 2.00 (2.00-2.00) |
| 51026 | 0 | — | — | 0 | — | — | 1 | 2.00 (0.00) | 2.00 (2.00-2.00) |
| 51059 | 209 | 2.38 (2.07) | 2.00 (0.00-3.00) | 402 | 3.66 (3.39) | 3.00 (1.00-5.00) | 222 | 1.82 (1.75) | 2.00 (1.00-2.00) |
| 51062 | 1 | 1.00 (0.00) | 1.00 (1.00-1.00) | 5 | 0.60 (0.55) | 1.00 (0.00-1.00) | 3 | 1.00 (1.00) | 1.00 (0.00-2.00) |
| 51067 | 0 | — | — | 0 | — | — | 6 | 2.17 (1.47) | 2.50 (1.00-3.00) |
| 51068 | 1 | 0.00 (0.00) | 0.00 (0.00-0.00) | 0 | — | — | 0 | — | — |
| 51072 | 0 | — | — | 1 | 0.00 (0.00) | 0.00 (0.00-0.00) | 1 | 0.00 (0.00) | 0.00 (0.00-0.00) |
| 51081 | 0 | — | — | 0 | — | — | 1 | 3.00 (0.00) | 3.00 (3.00-3.00) |
| 51082 | 5 | 3.20 (4.33) | 1.00 (0.00-5.00) | 11 | 1.18 (2.56) | 0.00 (0.00-1.00) | 19 | 1.21 (0.97) | 1.00 (0.00-2.00) |
| 51103 | 0 | — | — | 0 | — | — | 2 | 1.50 (0.71) | 1.50 (1.00-2.00) |
| 51104 | 0 | — | — | 1 | 6.00 (0.00) | 6.00 (6.00-6.00) | 1 | 1.00 (0.00) | 1.00 (1.00-1.00) |
| 51107 | 0 | — | — | 1 | 4.00 (0.00) | 4.00 (4.00-4.00) | 5 | 2.40 (0.55) | 2.00 (2.00-3.00) |
| 51126 | 0 | — | — | 2 | 2.00 (1.41) | 2.00 (1.00-3.00) | 2 | 3.00 (2.83) | 3.00 (1.00-5.00) |
| 51130 | 3 | 0.00 (0.00) | 0.00 (0.00-0.00) | 14 | 2.14 (1.53) | 2.00 (1.00-3.00) | 4 | 1.25 (1.50) | 1.00 (0.00-2.50) |
| 51149 | 0 | — | — | 0 | — | — | 3 | 0.33 (0.58) | 0.00 (0.00-1.00) |
| 51196 | 0 | — | — | 1 | 3.00 (0.00) | 3.00 (3.00-3.00) | 2 | 0.00 (0.00) | 0.00 (0.00-0.00) |
| 51201 | 0 | — | — | 4 | 4.00 (1.63) | 4.00 (3.00-5.00) | 0 | — | — |
| 51248 | 0 | — | — | 6 | 5.00 (4.56) | 4.50 (1.00-8.00) | 0 | — | — |
| 51249 | 0 | — | — | 3 | 2.67 (2.52) | 3.00 (0.00-5.00) | 0 | — | — |
| 51258 | 0 | — | — | 1 | 0.00 (0.00) | 0.00 (0.00-0.00) | 0 | — | — |
| 51265 | 0 | — | — | 0 | — | — | 1 | 7.00 (0.00) | 7.00 (7.00-7.00) |
| 51269 | 39 | 3.82 (2.57) | 4.00 (2.00-6.00) | 21 | 1.38 (2.29) | 1.00 (0.00-1.00) | 14 | 1.00 (1.04) | 1.00 (0.00-2.00) |
| 51298 | 2 | 0.50 (0.71) | 0.50 (0.00-1.00) | 3 | 5.67 (2.08) | 5.00 (4.00-8.00) | 5 | 0.60 (0.89) | 0.00 (0.00-1.00) |
| 51306 | 1 | 3.00 (0.00) | 3.00 (3.00-3.00) | 3 | 3.00 (1.73) | 4.00 (1.00-4.00) | 1 | 5.00 (0.00) | 5.00 (5.00-5.00) |
| 51321 | 1 | 0.00 (0.00) | 0.00 (0.00-0.00) | 0 | — | — | 2 | 3.50 (4.95) | 3.50 (0.00-7.00) |
| 51341 | 19 | 4.00 (3.02) | 4.00 (1.00-6.00) | 25 | 2.60 (2.61) | 2.00 (1.00-5.00) | 14 | 1.86 (1.79) | 1.00 (1.00-3.00) |
| 51343 | 5 | 2.20 (1.48) | 2.00 (2.00-3.00) | 26 | 1.58 (1.58) | 1.00 (0.00-2.00) | 21 | 1.48 (1.29) | 1.00 (1.00-2.00) |
| 51369 | 1 | 2.00 (0.00) | 2.00 (2.00-2.00) | 0 | — | — | 1 | 3.00 (0.00 | 3.00 (3.00-3.00) |
| 51394 | 1 | 0.00 (0.00) | 0.00 (0.00-0.00) | 3 | 1.00 (0.00) | 1.00 (1.00-1.00) | 12 | 1.17 (1.47) | 1.00 (0.00-2.00) |

*Values of 0 indicate that a user’s posts contributed no LDA-detected tokens; dashes indicate that a user did not post UGC relevant to the topic.
