# Supplementary material for: Integrating Natural Language Processing and Interpretive Thematic Analyses to Gain Human-Centered Design Insights on HIV Mobile Health: Proof-of-Concept Analysis
Source: JMIR Hum Factors. 2022 Jul 21;9(3):e37350. doi: 10.2196/37350 (PMC9353680; doi:10.2196/37350)
Supplement: Multimedia Appendix 3 [file humanfactors_v9i3e37350_app3.docx]

**Multimedia Appendix 3.** Human-detected intra-topic (Model 2) and intra-valence themes, with definitions and illustrative examples.

| Theme (*subtheme* in ital, if indicated) | Definition | Example* | Coding applications (*n*)† | |
| --- | --- | --- | --- | --- |
| **Topic A: Disease coping** | | | **Intra-topic** | **Extra-topic** |
| ART (*adherence*) | Descriptions and explorations of the role of ART *adherence* in users’ lives, including tips and personal histories | I have been completely adherent to taking my meds. Last night i got so caught up at my nephews welcome home dinner from a 6 day stay in the hospital that i forgot to take my medication. I know that missing 1 day is not likely to cause any changes in my VL or CD 4 levels but i feel awful that i missed a dose. | 17 | 1, B: Social adversities/“the voids in my life”  2, B: Social adversities/support networks  1, C: Salutations and check-ins/substance use and misuse |
| ART (*regimens*) | Descriptions and explorations of the role of specific ART *regimens* in users’ lives, including tips and personal histories | Anyone else have a doc who’s suggesting they change #meds for a simpler #regimen but isn’t quite ready to do it? […] A simpler regimen would be easier for #adherence and better for my heart and kidneys down the road so he’s suggesting I switch. | 6 | 0 |
| ART (*side-effects, interactions*) | Descriptions and explorations of the role of ART *side-effects and interactions* in users’ lives, including tips and personal histories | […] My answer is YES! I have had side effects from anything that contained AZT or its sister drugs or drugs that had it in it as a combo. I avoid AZT like the plague. | 9 | 0 |
| Raising awareness | Instances in which the Thrive With Me forum was used to share events, resources, and information | Please join us… for World AIDS Day 2017, to remember those we’ve lost and to raise our voices for health equity for all and an end to New York’s AIDS epidemic by 2020. FREE & OPEN TO ALL! | 23 | 2, B: Social adversities/“the voids in my life”  1, B: Social adversities/support networks  1, C: Salutations and check-ins/“other days I move mountains”  2, C: Salutations and check-ins/substance use and misuse  1, (–) Neg/mental health challenges  1, (–) Neg/mental health challenges |
| Disclosing serostatus | Experiences of, and perspectives on, disclosing  one’s HIV seropositivity in social, sexual, romantic, and professional settings | HIV disclosure is never easy, but I have learned from my own experiences the longer I’ve waited the harder it became to open up about my status. My remedy was to disclose the minute I know their is a potential for something more. | 14 | 1, B: Social adversities/“the voids in my life”  7, B: Social adversities/partnering challenges  1, B: Social adversities/support networks  1, B: Social adversities/trust and betrayal |
| Survivorship | Descriptions and explorations of long-term survival as sexual minority men living with HIV | Any fellow long term survivors here? This really touched me... Only when i see things like this do i kind of realize i on some level try NOT to think too deeply about the overall thoughts and feelings abt what i've been thru, seen close friends go thru and experience, and the losses of the last 25 years […] | 12 | 1, B: Social adversities/support networks |
| System contacts | Instances in which Thrive With Me forum users recounted specific healthcare system encounters and offer tips and peer-to-peer support in navigating them | In my case, I had struggled like many struggle with long lines at pharmacies and impersonal pharmacy staff and borderline violations of privacy in front of random customers at drugstores. That, of course, made me seriously consider getting my meds delivered. But a previous doctor suggested I ditch the chain stores and look for a good mom-and-pop pharmacy. That was the best move I ever made. | 8 | 3, B: Social adversities/support networks  1, C: Salutations and check-ins/substance use and misuse |
| Un/detectability | Descriptions and explorations of maintaining an undetectable viral load as a person living with HIV | Hey guys, I was reading one of the thrive tips about undetectable viral load and HIV stigma and I wanted to pose this question to you guys:do you think coming out as undetectable is better than coming out as HIV positive? | 11 | 0 |
| **Topic B: Social adversities** | | | **Intra-topic** | **Extra-topic** |
| Partnering challenges | Experiences of, and perspectives on, the barriers  to sexual and romantic partnering faced by sexual minority MSM living with HIV | Hang in there all relationships have that give and take and sometimes it can be hard cause you may feel at times you are taking to much. If you can take the HIV status of you both out of the picture and just deal with the love this guy is showing you may open up more room for a comforting relation. […] | 43 | 7, A: Disease coping/disclosing serostatus  4, C: Salutations and check-ins/substance use and misuse  1, (–) Neg/mental health challenges |
| Self-confidence | Descriptions of the role self-confidence can exert when navigating interpersonal obligations for sexual minority MSM living with HIV | Just be you! Most people can appreciate a person who is comfortable in their skin. Others might be intimidated by the level of comfort one has within themselves. It’s not always easy to accept what you don't understand but those issues are not your problem. […] | 3 | 0 |
| Support networks | Experiences of, and perspectives on, the role of social support networks for  sexual minority MSM living with HIV | Hello TWM Family, It’s been another challenging, moving forward week, and getting through it with the sense of accomplishment. […] take advantage of this site and the suggestions members post. The community suggestions with their renewed purpose, have inspired me to take action, thus improving the quality of my life. TWM members, lets continue too save a life, including our own. | 22 | 2, A: Disease coping/ART (*adherence*)  1, A: Disease coping/raising awareness  1, A: Disease coping/disclosing serostatus  3, A: Disease coping/system contacts |
| Trust and betrayal | Descriptions of trust, distrust, and broken trust, as a dimension of romantic and sexual partnering | I was dating someone who wanted an open relationship and I couldn’t do it. Mainly because the communication wasn’t there to even go there and we couldn’t agree on safer sex practices. I had told him I would feel better if I at least knew he would be safe, but he refused. But overall I’m not for it for me, but I do know plenty of couples that have that open communication and trust and it works great for them. | 15 | 1, A: Disease coping/disclosing serostatus  1, C: Salutations and check-ins/substance use and misuse |
| “the voids in my life” ‡ | Descriptions of unmet socio-structural needs, including expressions of isolation and loneliness | Since my elders has all gone to a peaceful place in heaven I have been lost in this world, and my generation has proven I have no one to love me as we don’t get along, and they do malicious thing to hurt me yet I still try hard to think and do positive things for others as a distraction from my own feelings and situation | 13 | 1, A: Disease coping/ART (*adherence*)  2, A: Disease coping/raising awareness  1, A: Disease coping/disclosing serostatus |
| **Topic C: Salutations and check-ins** | | | **Intra-topic** | **Extra-topic** |
| “other days, I move mountains” ‡ | Descriptions of recent personal and professional triumphs | I’m feeling pretty good and healthy today. i went to the gym a couple of hours ago and I feel great. my mood is always boosted after a good workout. it makes me feel like I can live with HIV, without any fear. | 10 | 1, A: Disease coping/raising awareness  1, B: Social adversities/support networks |
| Substance use and misuse | Descriptions of the roles alcohol and illegal drugs play (including via recovery) in the lives of Thrive With Me users | Tough weekend. I don’t consider myself an alcoholic, but I am a binge drinker and had a drink that turned into 5 and forgetting to take my pill. Then having a migraine for the rest of the weekend. Can anyone relate? Ugh, I get so hard on myself. It seems like when I am having a good week or a busy week I feel the need to release energy or escape by having a few drinks. […] Here’s looking to a better week ahead. | 21 | 1, A: Disease coping/ART (*adherence*)  2, A: Disease coping/raising awareness  1, A: Disease coping/system contacts  4, B: Social adversities/partnering challenges  7, B: Social adversities/support networks  1, B: Social adversities/trust and betrayal  1, (–) Neg/mental health challenges |
| **(–) Negative sentiment** | | | **Intra-valence** | **Extra-valence** |
| Reacting to media | Instances in which the Thrive With Me forum was used to share emotionally arousing external news media | You gave him life but you don't have the right to take his life away...... Rot in hell http://www.independent.com […] “Man allegedly shot and killed his own 14-year-old son for being gay A father allegedly shot dead his gay teenage son over his sexual orientation.” | 54 | 0 |
| Mental health challenges | Experiences of, and perspectives on, the specifically, explicitly invoked mental health challenges encountered by Thrive With Me users | not having a good week between being depress and isolating feel like im going crazy | 23 | 1, A: Disease coping/raising awareness  1, B: Social adversities/partnering challenges  1, C: Salutations and check-ins/substance use and misuse |
| Political climate | Emotional response on and speculation concerning the U.S. political climate during the Thrive With Me trial | I must vent. This entity that has been elected is causing so much division and turmoil. It scares me to the core and i cannot fathom how people whom support this madness can sleep at night. I have medical issues and to be honest they are the least of my worries. Im in disgust that our liberties are being torn away with the stroke of a pen. Horrified actually. We must fight this tooth and nail. Thoughts? | 16 | 1, A: Disease coping/raising awareness |
| **(+) Positive sentiment** | | | **Intra-valence** | **Extra-valence** |
| Gratitude | Expressions of gratitude, broadly considered | Thanks friends. Your support is crucial. Thank you | 41 | 0 |

*Examples are lightly edited for concision. Eccentricities in syntax are preserved.

†Intra-topic = a theme’s coding applications within the condensed UGC of the topic in which it was first detected; extra-topic = a theme’s coding applications within the condensed UGC of other topics or valences; intra-valence = a theme’s coding applications within the condensed UGC of the sentiment polarity in which it was first detected; extra-topic = a theme’s coding applications within the condensed UGC of other valences or topics.

‡*In vivo* code.
